# Indigenous knowledge and leadership for climate change adaptation in nutrition

**DOI:** 10.1371/journal.pgph.0003917

**Published:** 2024-11-14

**Authors:** Carol Zavaleta-Cortijo, Rosa Silvera-Ccallo, Guillermo Lancha-Rucoba, Junior Chanchari, Nerita Inuma, Manuel Pizango, Valeria Morales-Ancajima, Marianella Miranda-Cuadros, Juan-Pablo Aparco, Andrea Valdivia-Gago, Rocilda Nunta-Guimaraes, Teresita Antazú, Jorge Velez-Quevedo, Connie Fernandez-Neyra, Cesar Carcamo, Darren Greenwood, Janet Cade, James D. Ford, Sherilee Harper, J. Jaime Miranda

**Affiliations:** 1 Intercultural Citizenship and Indigenous Health Unit (UCISI), Cayetano Heredia Peruvian University (UPCH), Lima, Perú; 2 Community of Nuevo Progreso, Shawi Indigenous People, Loreto, Perú; 3 Community of Palmiche, Shawi Indigenous People, Loreto, Perú; 4 Community of 10 de Agosto, Shawi Indigenous People, Loreto, Peru; 5 National Center for Food and Nutrition (CENAN), National Instituto of Health (INS),Lima, Perú; 6 Indigenous Woman Leader, Shipibo-Konibo Indigenous People, Ucayali, Peru; 7 Indigenous Woman Leader, Yanesha Indigenous People, Pasco, Perú; 8 Taller Verde, Loreto, Perú; 9 Santa Gema Hospital, Red de Salud Alto Amazonas, Loreto, Perú; 10 School of Public Health and Administration, Cayetano Heredia Peruvian University (UPCH), Lima, Perú; 11 School of Medicine, University of Leeds, Leeds, United Kingdom; 12 Nutritional Epidemiology Group, University of Leeds, Leeds, United Kingdom; 13 Priestley Centre for Climate Futures, University of Leeds, Leeds, United Kingdom; 14 School of Public Health, University of Alberta, Edmonton, Canada; 15 Sydney School of Public Health, University of Sydney, Camperdown, Australia; 16 CRONICAS Center of Excellence in Chronic Diseases, Universidad Peruana Cayetano Heredia, Lima, Perú; PLOS: Public Library of Science, UNITED STATES OF AMERICA

## Indigenous knowledge and leadership for climate change adaptation in nutrition

Climate change adaptation to support our food and nutrition system will berequired to achieve sustainable development, especially in combating hunger (SDG 2) while also achieving health and mitigating climate change (SDG 3 and 13) [1]. Climate change repercussions for nutrition are especially concerning in countries where essential needs remain unmet [2]. For Indigenous and non-Indigenous scientists working in Latin America (LA), this means that we need to embrace positive transformationsto shape our future.Persistent health challenges that are embedded in social and structural inequities(e.g. prevalent infectious diseases [3], and high levels of under nutrition and anaemia [4]) are at risk of being exacerbated by climate change. For example,anaemia prevalence ranges from 16% to 86%, among young Indigenous children, with stunting and overweight affecting up to 48% and 40%, respectively of children in LA [4, 5].

Promisingly, the good news for LA countries,where up to 8% of its population self-identifies as Indigenous [6], is that we can recognise, preserve, and leverage the insights and knowledge of Indigenous Peoples. A reciprocal collaboration is essential to inform adaptation policies that ensureimproved nutrition for Indigenous communities whilst simultaneously supporting the sustainable development of countries by reducing harm to the environment, preserving cultures, and strengthening well-being and income opportunities for Indigenous Peoples.

## The research program

In 2022, the BioKusharu investigation was implemented in Peru by a team of Indigenous and non-Indigenous researchers as well as community members,and sought to document and characterisethe diet Indigenous Peoples consumed to increase climate change resilience and adaptation to extreme flooding [7]. The study was conducted in Balsapuerto, a Shawi territory in the Peruvian Amazon. Community members identified food insecurity as one of the health impact pathways of climate change, a concern that emerged during a previous collaboration [8]. A 24-hour food intake recallwas applied, using Shawi language and was adapted to recognise details like what specific type of plant, bird, fish, insect, or other food was consumed the day before the interview. Pictures were taken in the farm or home garden when possible. More than 160 species of food items were listed as part of that work [9].

## Ethics statement

The BioKusharu study was revised by the Peruvian ethics board of Universidad Peruana Cayetano Heredia register N° 104343; all participants have provided written informed consent to be part of the investigation.

One novel surprise was that families consumed many types of tubers that we, as Peruvians and Shawi researchers, were unaware of. Up to ten different species of tubers were consumed in Shawi´s diet. In Peru, *Yuca* (*Manihot esculenta* Crantz) is the typical tuber produced in the Amazon, and most tubers consumed are potatoes produced in the Andes, not in the Amazon. Finding that different species of tubers were consumed triggered the curiosity to know more about the production and nutrient content of these species. **Fig A**in **S1 Fig**, shows some of the species identified. We learned that mothers were the dedicated people whoplant and harvest these species. From Shawi Indigenous Peoples perspectives, women who are mothers, produce these tubers to feed their family.Grandmothers had particular species in their small farm that younger mothers did not have, making us speculate that there may be a risk that certain tuber seeds might not be passed down to the next generation.

We noted that communities were adapting to changing climatic conditions in the Amazon. One participant stated that she had changed the place where she was planting a tuber called *Uyuwan*tohigher landssince past flooding destroyed their crops, and she had to ask her sister to share the seeds or “*papitas*” in Spanish. Reflecting on the importance of tubers for nutritional adaptation, we also learned that these unique species arepromptly re-plantedand carefully cared for one year; they may not be conserved because of the warm temperatures prevalent in the rainforest, and these tubers are easily spoiled at ambient conditions. There is no access to refrigeration in the Shawi territory for a typical household, which again introduces the risk that some of these species may be lost in the future.

Having documented these unique species of tubers, we wanted to know theirnutritional contentin food composition tables to inform community members.

Although we found that Peruvian composition tables were incomplete for some nutrients, we also discovered that certain species contain higher levels of micronutrients, surpassing the nutritional content in*Papa amarilla (Solanum phureja)*,the most common type of tuber consumed in urban areas.For example, **Table A** in **S1 Table** shows *Pituca*(*Colocasia esculenta****)*** has more iron content than *Papa amarilla (Solanum phureja)*, 1.2 mg vs 0,4 mg per 100 g of edible food, respectively. In S1 Table we can also observe that the dietary fibre content in *Yuca* and *Pituca* is higher than that reported for *Papa amarilla*. Dietary fibre is a necessary nutrient to maintain good digestive and metabolic health; therefore, measuring its intake is important for a proper assessment of nutritional health. Additionally, *Pituca* has a higher concentration of vitamin A compared to *Papa amarilla* and *Yuca*. Vitamin A is essential for variousphysiological processes, immune system support, growth and development, making its consumption important for preventing nutritional deficiencies.

## The climate change adaptation

**Fig 1:**
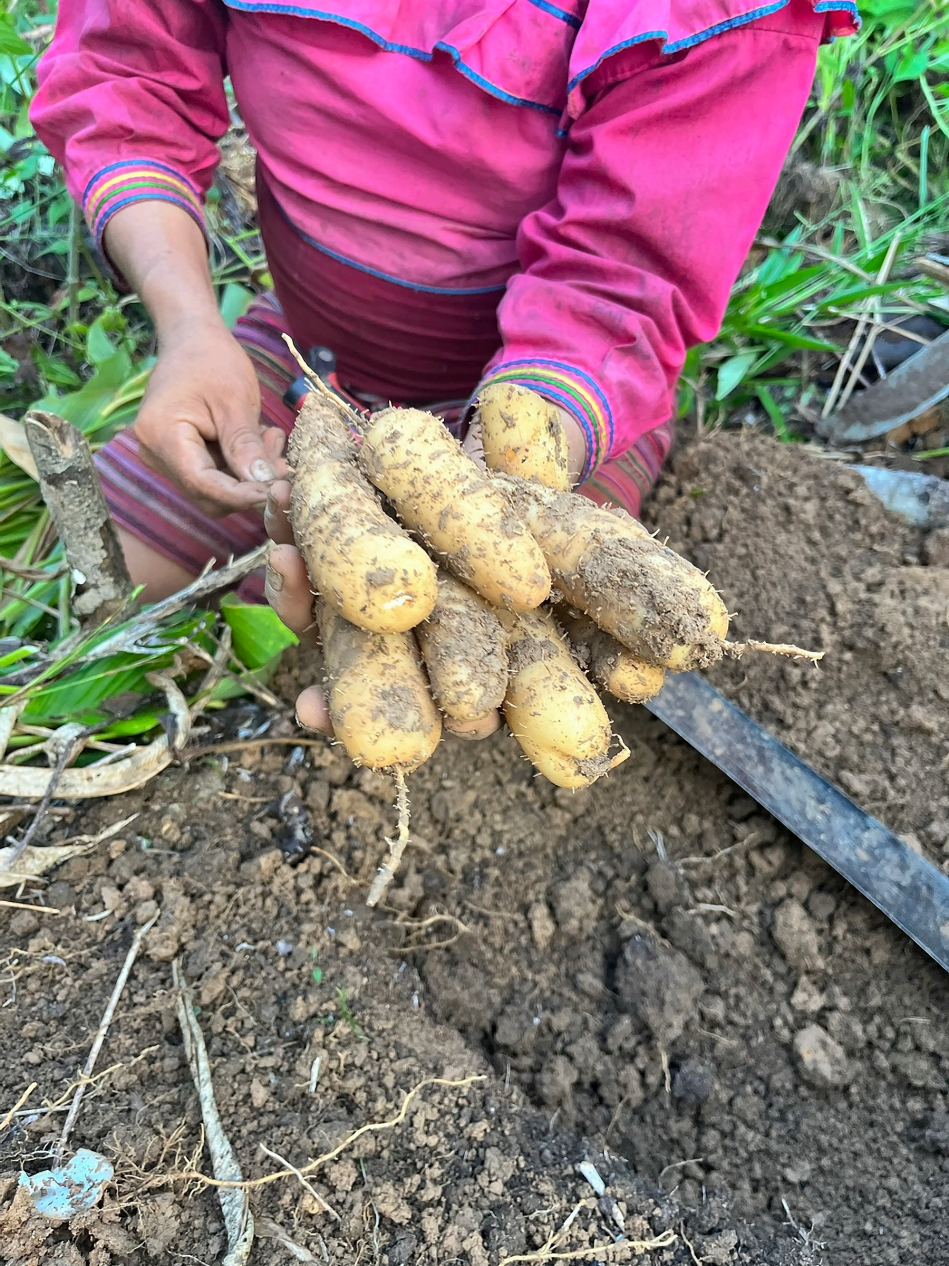
One variety of tuber harvested in the Shawi farm.

In this study, Shawi researchers suggestedinitiating a community farm to “*rescue*, *value*, *protect*, *store*, *use*, *plant and reproduce*” these culturally essential food species for increase the resilience to climate change. A community farm could be used as an educative space for translating information about Shawi tubers from parents to children, and to share the seeds among all the women who could participate in this activity. Shawi researchers also suggested that, in parallel, it would be important to explore the connections with private enterprises in the Peruvian gastronomic market. This connection will allow the community members to take advantage of the specialized *cousin* to provide global recognition and a better economic value for selling Amazonian tubers. The aspiration is that by identifyinga socially, culturally, and environmentally respectful and fair market, this adaptation will allow just economic compensation for the women and mothers who potentially engage in tuber production. Moreover, since Shawi methods do not use agrochemicals, seed and soil conservation is possible, contributing to climate-resilient food systems and sustainable diet. Fig 1 shows one variety of tuber harvested in the Shawi farm.

## Way forward

Recognition and preservation of Indigenous knowledge for climate change adaptation are essential not only in Peru or Latin America, but globally. Indigenous cosmologiesemphasize that food is more than only nutrients and is deeply connected to biocultural values, health and well-being [10].While mainstream science can contribute methodologies, technologies and ethical frameworks, such as identifying the nutrient content of diverse species, this must be done with full respect for Indigenous rights [11].This implies that the community’s knowledge must be preserved and valued, enabling them to take the lead as stewards in any food innovation process.

Indigenous food systems and knowledges play a crucial role and offer unique opportunities in adaptation to climate change while safeguarding nutrition, restoring the land, and protecting health. The co-creation of knowledge between Indigenous and non-Indigenous scientists, filled with mutual respectful and a humble curiosity to learn from community members, may trigger the innovation climate change nutritional adaptation requires. To fight hunger and malnutrition, food policies must embrace Indigenous knowledge and perspectives on the vital relationship between nutrition and Mother Nature for a sustainable future.

The Spanish version of this manuscript is in **S1 Text.**

## Supporting information

S1 Fig Picture shows some of the ten species of tubers identified by Shawi community members who participated in the BioKusharu study in the Peruvian Amazon. (TIFF)

S1 Table Table shows the different nutrient content of a Peruvian typical potato compared with Shawi Amazonian tubers. (DOCX)

S1 Text Spanish translation. (DOCX)

## References

[1] Miranda JJ, Zavaleta-Cortijo C. The food crisis in the context of climate change and sustainable development goals. Revista Peruana de Medicina Experimental y Salud Pública. 2023;40(4). doi: 10.17843/rpmesp.2023.404.13553 38597466 PMC11138824

[2] Cissé G, McLeman R, Adams H, Aldunce P, Bowen K, Campbell-Lendrus D, et al. Health, Wellbeing, and the Changing Structure of Communities. 2022 [cited Jan 31 2024]. In: Climate Change 2022: Impacts, Adaptation and Vulnerability Contribution of Working Group II to the Sixth Assessment Report of the Intergovernmental Panel on Climate Change [Internet]. Cambridge, UK and New York, NY, USA,: Cambridge University Press, [cited Jan 31 2024]; [1041–170]. Available from: https://www.ipcc.ch/report/ar6/wg2/chapter/chapter-7/.

[3] Tidman R, Abela-Ridder B, de Castañeda RR. The impact of climate change on neglected tropical diseases: a systematic review. Transactions of The Royal Society of Tropical Medicine and Hygiene. 2021;115(2):147–68. doi: 10.1093/trstmh/traa192 33508094 PMC7842100

[4] Rosas Jiménez CA, Tercan E, Horstick O, Igboegwu E, Dambach P, Louis VR, et al. Prevalence of anemia among Indigenous children in Latin America: a systematic review = Prevalencia de anemia en niños indígenas en Latinoamérica: una revisión sistemática. Rev Saude Publica. 2022;56. 10.11606/s1518-8787.2022056004360 PMC9749659 36515311

[5] Corvalán C, Garmendia M, Jones‐Smith J, Lutter C, Miranda JJ, Pedraza L, et al. Nutrition status of children in Latin America. Obesity reviews. 2017;18:7–18. doi: 10.1111/obr.12571 28741907 PMC5601284

[6] CEPAL. Indigenous Peoples in Latin America 2014 [cited 2024 Sep 30]. Available from: https://www.cepal.org/en/infografias/los-pueblos-indigenas-en-america-latina#:~:text=By%20the%20year%202010%2C%20an,special%20regulations%20for%20this%20purpose.

[7] Zavaleta-Cortijo C, Cade J, Ford J, Greenwood DC, Carcamo C, Silvera-Ccallo R, et al. Does food biodiversity protect against malnutrition and favour the resilience to climate change-related events in Amazon Indigenous communities? A protocol for a mixed methods study. Wellcome Open Res. 2022;7:246. Epub 20230626. doi: 10.12688/wellcomeopenres.18235.1 ; PubMed Central PMCID: PMC10924752. 38463717 PMC10924752

[8] Hofmeijer I, Ford JD, Berrang-Ford L, Zavaleta C, Carcamo C, Llanos E, et al. Community vulnerability to the health effects of climate change among indigenous populations in the Peruvian Amazon: a case study from Panaillo and Nuevo Progreso. Mitigation and Adaptation Strategies for Global Change. 2013;18:957–78.

[9] Silvera-Ccallo R, Tangoa NI, Pizango-Inuma R, Pizango Tangoa M, Huiñapi JC, Lancha-Rucoba G, et al. Extended Data 3 and 4: Lista de alimentos Shawi basado en la revision de varones y mujeres / Shawi food list according with men and according with women. figshare; 2023.

[10] FAO. The White/Wiphala Paper on Indigenous Peoples’ food systems. Rome2021.

[11] UN. United Nations Declaration on the Rights of Indigenous Peoples Act (UNDRIP) 2007 Sep 29 2024 [cited 2024 Sep 29]. Available from: https://www.un.org/development/desa/indigenouspeoples/wp-content/uploads/sites/19/2018/11/UNDRIP_E_web.pdf.

