# Supplementary figures and images for: Indigenous knowledge and leadership for climate change adaptation in nutrition
Source: PLOS Glob Public Health. 2024 Nov 14;4(11):e0003917. doi: 10.1371/journal.pgph.0003917 (PMC11563436; doi:10.1371/journal.pgph.0003917)

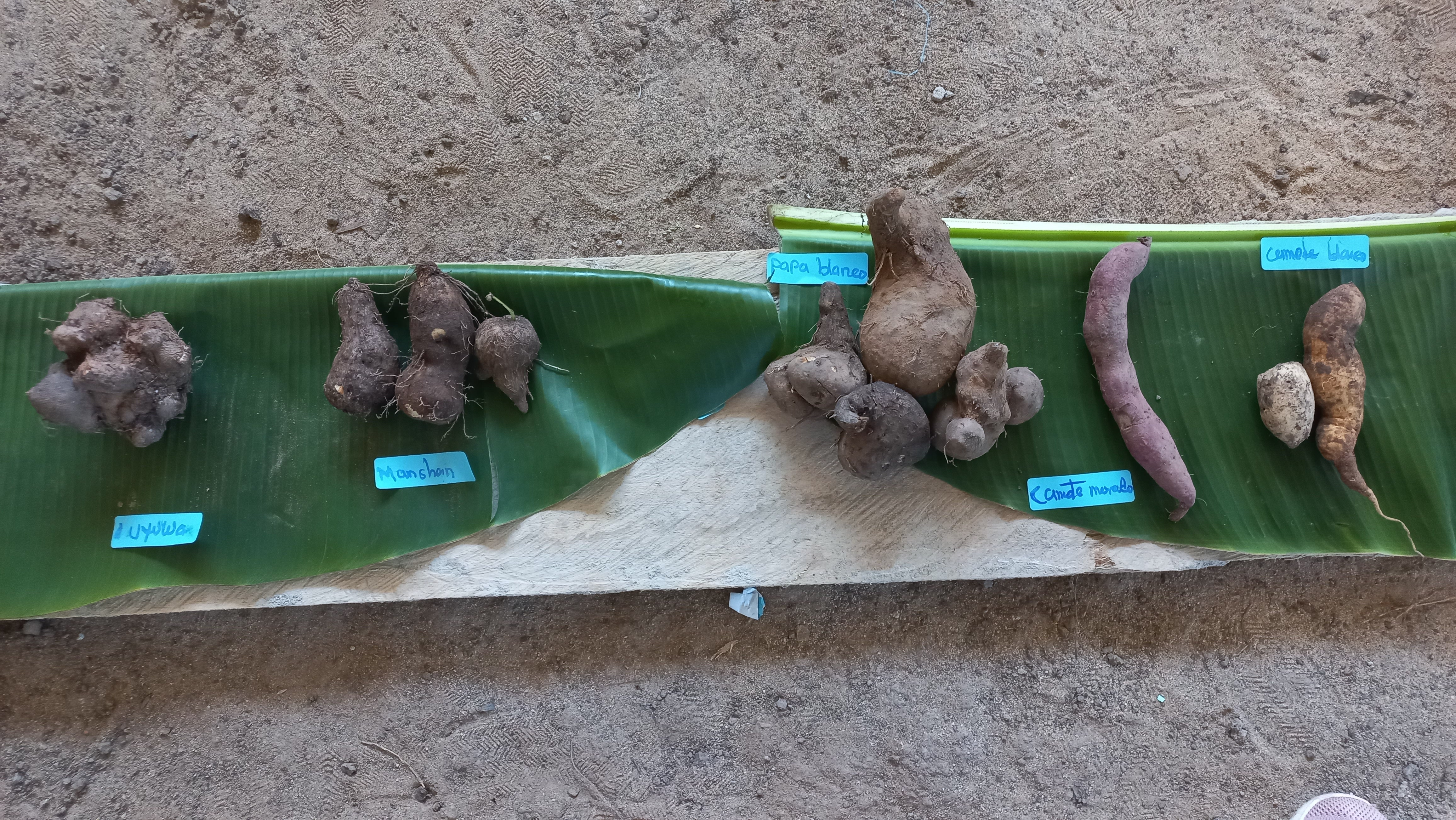

Supplement: S1 Fig — (TIFF) [file pgph.0003917.s001.tiff]
