# Supplementary material for: Indigenous knowledge and leadership for climate change adaptation in nutrition
Source: PLOS Glob Public Health. 2024 Nov 14;4(11):e0003917. doi: 10.1371/journal.pgph.0003917 (PMC11563436; doi:10.1371/journal.pgph.0003917)
Supplement: S1 Table — (DOCX) [file pgph.0003917.s002.docx]

| Per 100 g of edible part | Potato:  *Papa amarilla*  (*Solanum phureja)[1]*  # | Cassava:  *Yuca blanca*  (*Manihot esculenta*)[1]  * | Amazonian tuber:  *Pituca*  (*Colocasia esculenta*)[1]  * | Amazonian tuber*:*  *Dale Dale*  (*Goeppertia allouia)[1]*  * | Amazonian tuber:  *Sacha papa morada*  (*Dioscorea trifida*. var púrpura)[2]  * | Amazonian tuber:  Sacha papa blanca  (*Dioscorea trifida*. var blanco)[1]  * |
| --- | --- | --- | --- | --- | --- | --- |
| Energy (kcal) | 101 | 115 | 82 | 115 | 112 | 126 |
| Water (g) | 73,2 | 65,8 | 73,7 | 67,0 | 72,2 | 67,2 |
| Protein (g) | 2,0 | 0,7 | 1,6 | 4,3 | 1,8 | 2,5 |
| Fat (g) | 0,4 | 0,5 | 0,5 | 0,2 | 1,8 | 0,1 |
| Carbohydrates totals (g) | 23,3 | 31,8 | 23,2 | 26,5 | 23,5 | 29,4 |
| Fibre (g) | 0,4 | 3,5 | 4,1 | sd | 0,4 | sd |
| Calcium (mg) | 6 | 25 | 50 | 147 | 3 | 4 |
| Vitamina A ( ug) | 0 | 0 | 4 | sd | sd | sd |
| Phosporo (mg) | 52 | 52 | 41 | 104 | 30 | 38 |
| Zinc (mg) | 0,3 | 0,63 | 0,23 | sd | sd | sd |
| Iron (mg) | 0,40 | 0,19 | 1,20 | 0,80 | 0,7 | 0,60 |

**Table 1**: This table was made with the following references[1, 2] to show the different nutrient content of a Peruvian typical potato (#) compared with Shawi Amazonian tubers (*). sd: no data

1. Reyes Garcia M. Tablas peruanas de composición de alimentos/Elaborado por María Reyes García; Iván Gómez-Sánchez Prieto, Cecilia Espinoza Barrientos. Lima: Ministerio de Salud, Instituto Nacional de Salud,, 2017.

2. Astete Verde KS. Sustitución parcial de harina de trigo (Triticum aestivum L.) por la mezcla de harina de Sachapapa Morada (Dioscórea trífida L.) y harina de soya (Glycine max L.) en la elaboración de panes en Pucallpa. Pucallpa, Peru: Universidad Nacional de Ucayali; 2019.
