# Supplementary material for: Indigenous knowledge and leadership for climate change adaptation in nutrition
Source: PLOS Glob Public Health. 2024 Nov 14;4(11):e0003917. doi: 10.1371/journal.pgph.0003917 (PMC11563436; doi:10.1371/journal.pgph.0003917)
Supplement: S1 Text — (DOCX) [file pgph.0003917.s003.docx]

**Statement of Responsibility:** This translation has not been reviewed by the journal

**Declración de responsabilidad:** Esta traduccion no ha sido revisada por la revista

**Conocimientos y Liderazgo Indígena para la Adaptación al Cambio Climático en la Nutrición**

La adaptación al cambio climático de nuestro sistema alimentario y nutricional es necesaria a fin de lograr un desarrollo sostenible, especialmente en la lucha contra el hambre (ODS 2), al tiempo que se logra la salud y se mitiga el cambio climático (ODS 3 y 13).[1]Las repercusiones del cambio climático en la nutrición son especialmente preocupantes en los países donde las necesidades esenciales siguen sin satisfacerse.[2]Para los científicos Indígenas y no Indígenas que trabajan en América Latina (AL), esto significa que debemos adoptar transformaciones positivas para definir nuestro futuro. Los desafíos de salud persistentes arraigados en las desigualdades sociales y estructurales (por ejemplo, las enfermedades infecciosas prevalentes)[3], y altos niveles de desnutrición y anemia[4]) corren el riesgo de agravarse frente a los impactos del cambio climático. Por ejemplo, la prevalencia de anemia oscila entre el 16% y el 86% entre los niños Indígenas, mientras que el retraso del crecimiento y el sobrepeso afectan hasta el 48% y el 40%, respectivamente, de los niños de América Latina.[4, 5].

Una buena noticia para los países de América Latina, donde hasta el 8% de su población se autoidentifica como Indígena.[6], es que podemos reconocer, preservar y aprovechar las ideas y el conocimiento de los pueblos Indígenas. Sin embargo, una colaboración en reciprocidad es esencial para generar políticas de adaptación que garanticen una mejor nutrición para las comunidades Indígenas y, al mismo tiempo, respalden el desarrollo sostenible de los países mediante la reducción del daño al medio ambiente, la preservación de las culturas y el fortalecimiento del bienestar y las oportunidades de ingresos económicos para los pueblos Indígenas.

**El programa de investigación**

En 2022, la investigación [BioKusharu](https://www.biokusharu.com/) fue implementada en Perú por un equipo de investigadores Indígenas y no-Indígenas, así como miembros de la comunidad, y buscó documentar y caracterizar la dieta que consumían los pueblos Indígenas para aumentar la resiliencia al cambio climático y la adaptación a las inundaciones extremas.[7]El estudio se llevó a cabo en Balsapuerto, un territorio Shawi en la Amazonía peruana. Los miembros de la comunidad identificaron la inseguridad alimentaria como una de las vías de impacto del cambio climático en la salud, una preocupación que surgió durante una colaboración previa.[8]Se aplicó un recordatorio de la ingesta de alimentos de 24 horas, en idioma Shawi y adaptado para reconocer detalles como qué tipo específico de planta, pájaro, pez, insecto u otro alimento se consumió el día anterior a la entrevista. Se tomaron fotografías en la chacra o la huerta de la casa cuando fue posible. Se enumeraron más de 160 especies de alimentos como parte de ese trabajo.[9].

Una sorpresa fue que las familias consumían muchos tipos de tubérculos que nosotros, como peruanos e investigadores Shawi, desconocíamos. Hasta diez especies diferentes de tubérculos se consumían en la dieta de Shawi. En Perú, la Yuca (Manihot esculenta Crantz) es el tubérculo típico producido en la Amazonía, y la mayoría de los tubérculos consumidos en Perú son papas producidas en los Andes, no en la Amazonía. Encontrar que se consumían diferentes especies de tubérculos despertó la curiosidad por saber más sobre la producción y el contenido nutricional de estas especies. **Imagen 1** En la información complementaria 1, se muestran algunas de las especies identificadas. Aprendimos que las madres son las personas dedicadas que plantan, cuidan y cosechan estas especies. Desde la perspectiva de los pueblos Indígenas Shawi, las mujeres que son madres son las encargadas de producir estos tubérculos para alimentar a su familia. Las abuelas tenían en su pequeña chacra especies particulares que las madres más jóvenes ya no tenían, lo que nos hizo especular que puede haber un riesgo de que ciertas semillas de tubérculos no se transmitan a la siguiente generación.

Notamos que las comunidades se estaban adaptando a las condiciones climáticas cambiantes en la Amazonía. Una participante afirmó que había cambiado el lugar donde estaba plantando un tubérculo llamado Uyuwan a tierras más altas porque las inundaciones anteriores destruyeron sus cultivos, y tuvo que pedirle a su hermana que compartiera las semillas o “papitas” en español. Al reflexionar sobre la importancia de los tubérculos para la adaptación nutricional, también aprendimos que estas especies únicas se replantan rápidamente y se cuidan con dedicación durante un año, es posible que no se conserven debido a las temperaturas cálidas que prevalecen en la selva tropical, y estos tubérculos se estropean fácilmente en las condiciones ambientales. En el territorio Shawi, una familia típica no tiene acceso a refrigeración, lo que nuevamente introduce el riesgo de que algunas de estas especies se pierdan en el futuro.

Después de documentar estas especies únicas de tubérculos, queríamos conocer su contenido nutricional en tablas de composición de alimentos para informar a los miembros de la comunidad. Si bien encontramos que las tablas de composición peruanas estaban incompletas para algunos nutrientes, también descubrimos que ciertas especies contienen niveles más altos de micronutrientes, superando el contenido nutricional en *Papa amarilla (Solanum phureja),* el tipo de tubérculo común consumido en las zonas urbanas. Por ejemplo, la Tabla 1 de la Información complementaria 2 muestra Pituca (*Colocasia esculenta****).*** La yuca y la pituca tienen mayor contenido de hierro que la papa amarilla (Solanum phureja), 1,2 mg vs 0,4 mg por cada 100 g de alimento comestible, respectivamente. En la Tabla 1 también podemos observar que el contenido de fibra dietética en la yuca y la pituca es mayor que el reportado para la papa amarilla. La fibra dietética es un nutriente necesario para mantener una buena salud digestiva y metabólica; por lo tanto, medir su ingesta es importante para una evaluación adecuada de la salud nutricional. Además, la pituca tiene una mayor concentración de vitamina A en comparación con la papa amarilla y la yuca. La vitamina A es esencial para varios procesos fisiológicos, el apoyo al sistema inmunológico, el crecimiento y el desarrollo, por lo que su consumo es importante para prevenir deficiencias nutricionales.

**La adaptación al cambio climático**

En este estudio, los investigadores Shawi sugirieron iniciar una chacra comunitaria para “rescatar, valorar, proteger*, almacenar, usar, plantar y reproducir* “ Estas especies alimenticias culturalmente esenciales aumentan la resiliencia al cambio climático. Una chacra comunitaria podría ser usado como un espacio educativo para transmitir la información sobre los tubérculos Shawi de padres a hijos, y compartir las semillas entre todas las mujeres que pudieran participar en esta actividad. Los investigadores Shawi también sugirieron que, en paralelo, sería importante explorar las conexiones con empresas privadas en el mercado gastronómico peruano. Esta conexión permitirá a los miembros de la comunidad aprovechar la cocina especializada para brindar reconocimiento mundial y un mejor valor económico para la venta de tubérculos amazónicos. La aspiración es que, al identificar un mercado social, cultural y ambientalmente respetuoso y justo, esta adaptación permitirá una compensación económica justa para las mujeres y madres que potencialmente participan en la producción de tubérculos. Además, como los métodos Shawi no utilizan agroquímicos, es posible conservar las semillas y el suelo, lo que contribuye a la creación de sistemas alimentarios resilientes al clima y a una dieta sostenible.

**El camino a seguir**

El reconocimiento y la preservación del conocimiento Indígena para la adaptación al cambio climático son esenciales no solo en Perú o América Latina, sino a nivel mundial. Las cosmologías Indígenas enfatizan que los alimentos son más que solo nutrientes y están profundamente conectados con los valores bioculturales, la salud y el bienestar.[10].. Si bien la ciencia convencional puede aportar metodologías, tecnologías y marcos éticos, como la identificación del contenido de nutrientes de diversas especies, esto debe hacerse con pleno respeto por los derechos Indígenas.[11]. Esto implica que el conocimiento de la comunidad debe preservarse y valorarse, permitiéndoles tomar la iniciativa como lideres en cualquier proceso de innovación alimentaria.

Los sistemas alimentarios y conocimientos Indígenas desempeñan un papel crucial y ofrecen oportunidades únicas de adaptación al cambio climático, al tiempo que salvaguardan la nutrición, restauran la tierra y protegen la salud. La co-creación de conocimiento entre los científicos Indígenas y no Indígenas, llenos de respeto mutuo y de una humilde curiosidad por aprender de los miembros de la comunidad, pueden impulsar la innovación que requiere la adaptación nutricional al cambio climático. Para combatir el hambre y la malnutrición, las políticas alimentarias deben incorporar los conocimientos y las perspectivas indígenas sobre la relación vital entre la nutrición y la Madre Naturaleza para un futuro sostenible.


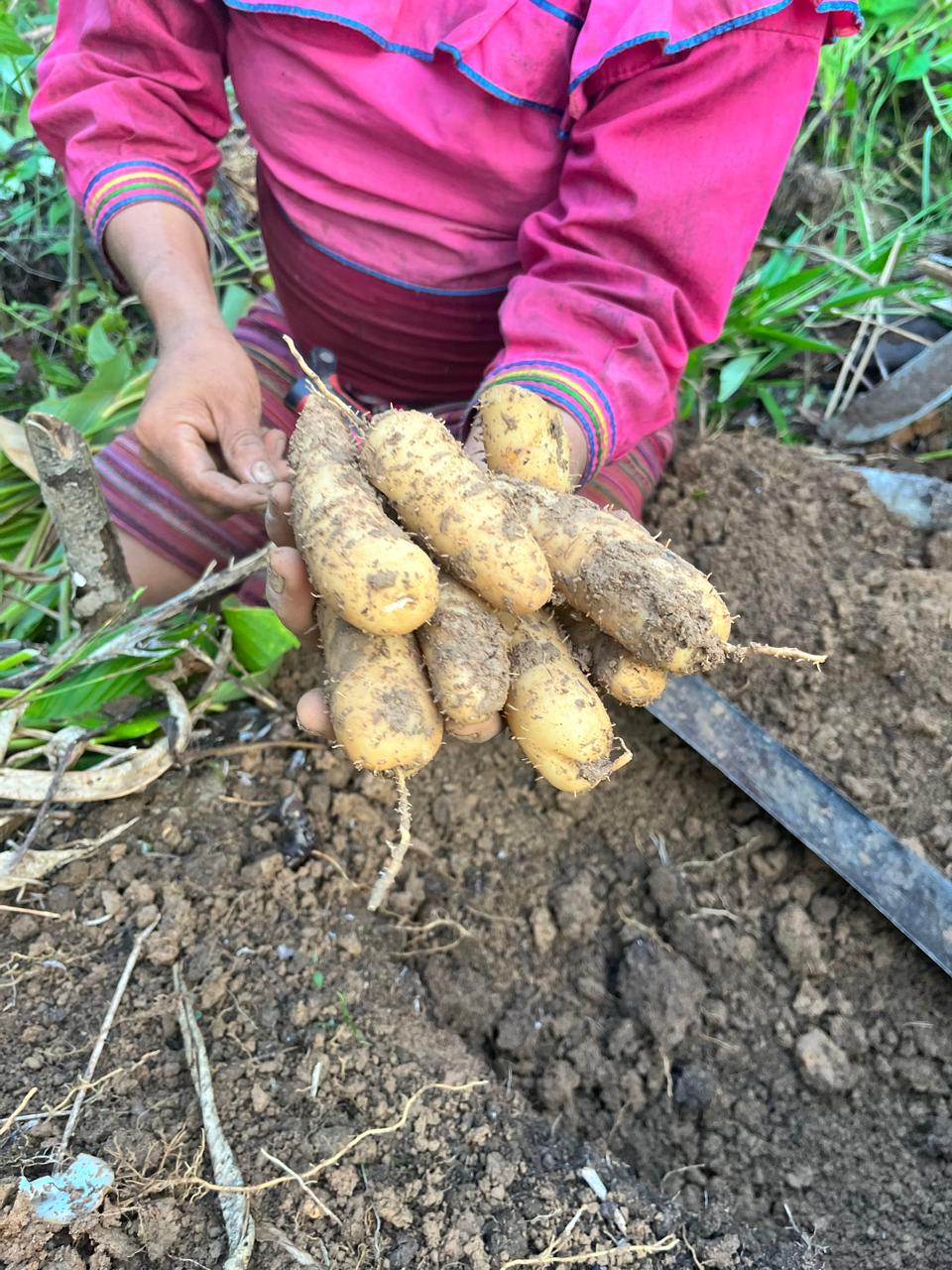


La figura 1 muestra una variedad de tubérculo cosechado en la granja Shawi.

**Declaración de divulgación financiera.**

El estudio BioKusharu fue financiado por el Instituto Nacional de Investigación en Salud (NIHR) (utilizando la financiación de la Asistencia Oficial para el Desarrollo (AOD) del Reino Unido) y Wellcome 218743_Z_19_Z en el marco de la Asociación NIHR-Wellcome para la Investigación en Salud Global. Las opiniones expresadas son las de los autores y no necesariamente las de Wellcome, el NIHR o el Departamento de Salud y Asistencia Social. Los financiadores no tuvieron ningún papel en el diseño del estudio, la recopilación y el análisis de datos, la decisión de publicar o la preparación del manuscrito.

**Intereses en competencia**

He leído la política de la revista y los autores de este manuscrito tienen los siguientes intereses en conflicto: CZ-C declara que su salario fue cubierto parcialmente con fondos de la Universidad de Alberta y la Universidad de Leeds mientras trabajaba en este manuscrito. Además, se identifica como una científica indígena de ascendencia quechua de los Andes del norte del Perú.

JC es director de Dietary Assessment Ltd, que apoya a myfood24. Además, dirige un centro colaborador de la OMS en epidemiología nutricional.

Otros autores no tienen intereses en competencia que declarar

**Expresiones de gratitud**

Al presentar esta información, queremos reconocer y honrar al territorio indígena Shawi y a sus miembros comunitarios, especialmente a los que habitan el río Armanayacu en el distrito de Balsapuerto, provincia del Alto Amazonas en la región Loreto, donde se impartieron estas experiencias y aprendizajes. De acuerdo con la autorización, reconocemos a la organización indígena Shawi Gobierno Territorial Autónomo de la Nación Shawi como colaboradora en la implementación del estudio BioKusharu.

Agradecemos a la Profesora Harriet V. Kuhnlein y a la Dra. Gina Kennedy por tomarse el tiempo de leer nuestro manuscrito y por sus valiosas sugerencias para mejorarlo.

**Declaraciones éticas**

El estudio BioKusharu fue revisado por el comité de ética peruano de la Universidad Peruana Cayetano Heredia registro N° 104343; todos los participantes han dado su consentimiento informado por escrito para ser parte de la investigación.

**Referencias**

1. Miranda JJ, Zavaleta-Cortijo C. La crisis alimentaria en el contexto del cambio climático y los objetivos de desarrollo sostenible. Revista Peruana de Medicina Experimental y Salud Pública. 2023;40(4). doi: 10.17843/rpmesp.2023.404.13553.

2. Cissé G, McLeman R, Adams H, Aldunce P, Bowen K, Campbell-Lendrus D, et al. Salud, bienestar y la estructura cambiante de las comunidades. 2022 [citado el 31 de enero de 2024]. En: Cambio climático 2022: impactos, adaptación y vulnerabilidad Contribución del Grupo de trabajo II al sexto informe de evaluación del Grupo Intergubernamental de Expertos sobre el Cambio Climático [Internet]. Cambridge, Reino Unido y Nueva York, NY, EE. UU.,: Cambridge University Press, [citado el 31 de enero de 2024]; [1041–170]. Disponible en:<https://www.ipcc.ch/report/ar6/wg2/chapter/chapter-7/>.

3. Tidman R, Abela-Ridder B, de Castañeda RR. El impacto del cambio climático en las enfermedades tropicales desatendidas: una revisión sistemática. Transactions of The Royal Society of Tropical Medicine and Hygiene. 2021;115(2):147-68. doi: 10.1093/trstmh/traa192.

4. Rosas Jiménez CA, Tercan E, Horstick O, Igboegwu E, Dambach P, Louis VR, et al. Prevalencia de anemia en niños indígenas en Latinoamérica: una revisión sistemática= Prevalencia de anemia en niños indígenas en Latinoamérica: una revisión sistemática. Rev Saude Publica. 2022;56.

5. Corvalán C, Garmendia M, Jones‐Smith J, Lutter C, Miranda JJ, Pedraza L, et al. Estado nutricional de los niños en América Latina. Obesity reviews. 2017;18:7-18.

6. CEPAL. Pueblos indígenas en América Latina 2014 [consultado el 30 de septiembre de 2024]. Disponible en:[https://www.cepal.org/es/infografias/los-pueblos-indigenas-en-america-latina#:~:text=By%20the%20year%202010%2C%20an,special%20regulations%20for%20this %20propósito](https://www.cepal.org/en/infografias/los-pueblos-indigenas-en-america-latina#:~:text=By%20the%20year%202010%2C%20an,special%20regulations%20for%20this%20purpose).

7. Zavaleta-Cortijo C, Cade J, Ford J, Greenwood DC, Carcamo C, Silvera-Ccallo R, et al. ¿La biodiversidad alimentaria protege contra la desnutrición y favorece la resiliencia a los eventos relacionados con el cambio climático en las comunidades indígenas amazónicas? Un protocolo para un estudio de métodos mixtos. Wellcome Open Res. 2022;7:246. Publicación electrónica 20230626. doi: 10.12688/wellcomeopenres.18235.1. PubMed PMID: 38463717; PubMed Central PMCID: PMCPMC10924752.

8. Hofmeijer I, Ford JD, Berrang-Ford L, Zavaleta C, Carcamo C, Llanos E, et al. Vulnerabilidad comunitaria a los efectos del cambio climático en la salud entre las poblaciones indígenas de la Amazonía peruana: un estudio de caso de Panaillo y Nuevo Progreso. Estrategias de mitigación y adaptación al cambio global. 2013;18:957-78.

9. Silvera-Ccallo R, Tangoa NI, Pizango-Inuma R, Pizango Tangoa M, Huiñapi JC, Lancha-Rucoba G, et al. Datos ampliados 3 y 4: Lista de alimentos Shawi basado en la revisión de varones y mujeres / Lista de alimentos Shawi según hombres y según mujeres. higuera; 2023.

10. FAO. Libro Blanco/Wiphala sobre los sistemas alimentarios de los pueblos indígenas. Roma 2021.

11. ONU. Ley de la Declaración de las Naciones Unidas sobre los Derechos de los Pueblos Indígenas (DNUDPI) 2007 29 de septiembre de 2024 [consultado el 29 de septiembre de 2024]. Disponible en:<https://www.un.org/development/desa/indigenouspeoples/wp-content/uploads/sites/19/2018/11/UNDRIP_E_web.pdf>.

**Información complementaria 1.**La imagen muestra algunas de las diez especies de tubérculos identificadas por los miembros de la comunidad Shawi que participaron en el estudio BioKusharu en la Amazonía peruana.

**Información complementaria 2.**La tabla muestra el diferente contenido de nutrientes de una papa típica peruana en comparación con los tubérculos amazónicos Shawi

**Información complementaria 3.**traducción al español
